# Enterovirus 71 viral capsid protein linear epitopes: Identification and characterization

**DOI:** 10.1186/1743-422X-9-26

**Published:** 2012-01-20

**Authors:** Fan Gao, Yi-Ping Wang, Qun-Ying Mao, Xin Yao, Shuang Liu, Feng-Xiang Li, Feng-Cai Zhu, Jing-Yu Yang, Zheng-Lun Liang, Feng-Min Lu, Jun-Zhi Wang

**Affiliations:** 1 National Institutes for Food and Drug Control, Beijing 100050, China; 2 ShenYang Pharmaceutical University, Shenyang 110016, China; 3 Beijing YouAn Hospital, Capital Medical University, Beijing 100069, China; 4 Jiangsu Provincial Center for Disease Prevention and Control, Nanjing 210009, China; 5 Peking University Health Science Center, Beijing 100191, China

**Keywords:** EV71, Capsid protein, Epitopes, Humoral immune response

## Abstract

**Background:**

To characterize the human humoral immune response against enterovirus 71 (EV71) infection and map human epitopes on the viral capsid proteins.

**Methods:**

A series of 256 peptides spanning the capsid proteins (VP1, VP2, VP3) of BJ08 strain (genomic C4) were synthesized. An indirect enzyme-linked immunosorbent assay (ELISA) was carried out to detect anti-EV71 IgM and IgG in sera of infected children in acute or recovery phase. The partially overlapped peptides contained 12 amino acids and were coated in the plate as antigen (0.1 μg/μl). Sera from rabbits immunized with inactivated BJ08 virus were also used to screen the peptide panel.

**Results:**

A total of 10 human anti-EV71 IgM epitopes (vp1-14 in VP1; vp2-6, 21, 40 and 50 in VP2 and vp3-10, 12, 15, 24 and 75 in VP3) were identified in acute phase sera. In contrast, only one anti-EV71 IgG epitope in VP1 (vp1-15) was identified in sera of recovery stage. Four rabbit anti-EV71 IgG epitopes (vp1-14, 31, 54 and 71) were identified and mapped to VP1.

**Conclusion:**

These data suggested that human IgM epitopes were mainly mapped to VP2 and VP3 with multi-epitope responses occurred at acute infection, while the only IgG epitope located on protein VP1 was activated in recovery phase sera. The dynamic changes of humoral immune response at different stages of infection may have public health significance in evaluation of EV71 vaccine immunogenicity and the clinical application of diagnostic reagents.

## Background

Human enterovirus 71 (EV71) is one of the major causative pathogens for human hand foot and mouth disease (HFMD). EV71 was first isolated in California in 1969. HFMD is common infectious disease frequently occurring in infants and children. Although it usually has no life threatening, the most severe neurological disease caused by EV71 may cause death. Therefore, EV71 is widely considered as one of the most important virulent neurotropic enteroviruses after the eradication of poliomyelitis [1].

During the last decade, outbreaks of HFMD have occurred worldwide and the incidence rate was significantly increased throughout the Asia-Pacific region [2-8]. The continuing increased HFMD epidemics in China over the last 3 years indicated HFMD has been an important public health concern [6-8]. Due to the absence of effective antiviral therapy for severe EV71 infections, the development of efficacious vaccine is a top priority to effectively prevent this disease.

EV71 is a picornavirus, belonging to the type A non-polio enteroviruses family [9]. It has a positive sense linear single stranded RNA genome of approximately 7,400 nucleotides, with a single open reading frame that encodes 4 capsid proteins (VP1-4). VP4 is located inside the virus capsid and connected tightly with the viral genome. Other three proteins (VP1-3) are exposed outside of the capsid and consequently immunologically reactive epitopes may reside in these proteins [10].

Previous studies using animal infectious models to develop vaccine found that majority of neutralizing antibody were elicited by the epitopes on VP1 protein [11-14]. Therefore, most relevant researches have focused on VP1 protein but neglecting the fact that VP2 and VP3 are also important components of EV71 virion. They play a role in eliciting immune responses during infection. Until now, no human epitope has been identified on VP2 and VP3 proteins. Usually, an acute viral infection should lead to certain epitope inducing an IgM response, which is a significant marker for early diagnosis of disease, as well as for the prompt treatment of the severe cases. However, almost all of the known epitopes on VP1 only elicit an IgG response.

To extend the range of defined EV71 epitopes, we employed a panel of synthetic peptides covering all three external capsid proteins (VP1-3) as antigen to screen IgM and IgG antibodies specifically for EV71 epitopes. The EV71 sera were obtained from infected children at different stages of infectious disease (the acute or recovery stage). Convalescent sera from rabbits were immunized with inactivated BJ08 strain EV71 virus.

## Materials and methods

### Virus antigen and antigen peptides synthesis

Virus particles of EV71 BJ08 strain isolated from throat swab samples of EV71 infected children in Beijing in 2008 [15] were inactivated as antigen for rabbit immunizations. The inactivated EV71 virus with 95% purity was kindly provided by the National Vaccine & Serum Institute (Beijing, China) at a concentration of 27.4 μg/ml.

A series of 256 overlapping peptides (each 12 amino acids with a 9 amino acid overlapping), were synthesized based on the deduced amino acid sequence of the VP1, VP2 and VP3 proteins of EV71 BJ08 strain. The purity of the synthesized peptides exceeded 70%. A peptide from the surface antigen of Hepatitis B virus (S_28-39 _- IPQSLDSWWTSL) was synthesized as a control. All peptides were resuspended in dimethyl sulfoxide at a concentration of 10 mg/ml stock solution at -20°C. A fresh working solution with a final peptide concentration of 0.1 μg/ml was prepared for coating antigen.

SPF grade New Zealand White female rabbits weighing 1.75-2.25 kg were used for experiments. All animals were purchased from Resource Center for Experimental Animals of the National Institute for Food and Drug Control, China.

### Human sera specimens

Eight anti EV71 sera were collected from infected children in acute stage of HFMD (defined as within 3 days after the appearance of the first symptoms). All sera samples were confirmed as EV71 virus positive by PCR detection of viral RNA [16], and were verified as no cross-reactivity for CA16 by microneutralization assay. Eleven recovery sera samples (defined as 2 month after illness onset) were also collected from recovered children. All sera samples were collected by CDC in Jiangsu Provence, China.

Sera from 4 healthy individuals with negative anti EV71 IgM and/or IgG (screened by microneutralization assay [17] and an *in vitro *indirect ELISA assay) were used as controls in this study.

### Viral RNA extraction and PCR amplification [16]

Viral RNA was extracted from clinical specimens using a QIAamp Mini viral RNA Extraction Kit (Qiagen) according to the manufacture's protocol. The primers used for RT-PCR are cited from reference 16. RT-PCR amplification was performed using AccessQuickTM RT-PCR kit (Promega). Cycle variables were reverse transcription at 45°C for 45 min, denaturation at 94°C for 5 min followed by 35 cycles at 95°C for 40 sec, 53°C for 40 sec, 72°C for 40 sec; and then a final elongation step at 72°C for 5 min. The second round amplification was performed in 25 μl volumes, which contains 2.5 μl 10 × PCR reaction buffer, 1 μl 10 mM dNTP, 0.5 μl 10 mM each primer, 0.5 μl Taq (TaKaRa), 1 μl of the first-round PCR product, and 19 μl Nuclease-Free Water, under the same conditions as the first-round PCR, except reverse transcription step. The PCR products were examined by electrophoresis with a 3.0% agarose gel.

### Preparation of rabbit anti EV71 serum

Rabbit blood collected before immunization was used to generate negative control sera. Equal volumes of heat-inactivated EV71 BJ08 strain and complete Freund's adjuvant were thoroughly mixed to produce an emulsion. Then the mixture of 3 ml was injected intramuscularly into the rear foot pads and inner thigh muscles of a rabbits. Boosting immunizations were administered at day 7, 14, 28 and 42 post primary immunizations using the same method with a exception of replacing Freunds's complete adjuvant with incomplete adjuvant. Fourteen days after the last immunization, blood was collected by cardiac puncture, and the sera were obtained and stored at -20°C.

### Indirect ELISA assay for detecting anti EV71 IgM and IgG

Purified EV71 viral particles were diluted to a final working concentration (0.01 μg/μl); meanwhile, the synthetic peptides were diluted to a final concentration of 0.1 μg/μl in carbonate buffer (0.1 M pH9.6). These antigen solutions were then coated separately into wells of a 96-well ELISA plate using 50 μl per well. The plate was kept at 4°C over night, washed once with phosphate buffered saline containing 0.05% Tween-20 (PBST), blocked for 2 hours with 100 μl per well of casein buffer. Then 50 μl of appropriately diluted serum was added to each well and incubated at 37°C for 30 min. The plate was then washed 5 times with PBST, followed by adding 50 μl horseradish peroxidase (HRP) conjugated goat anti-rabbit IgG or goat anti-human IgG or goat anti-human IgM (all diluted 1:40,000) and incubated for a further 30 min at 37°C, and the plate was wash for 5 times with PBST. For visualization, 100 μl of TMB substrate solution was added into each well and incubated at 37°C for 10 mins in a dark situation. The optical densities (OD) were measured at both 450 nm and 630 nm within 10 min after adding 50 μl 2 M sulfuric acid to stop the reaction. Cutoff value was calculated as mean OD value of the negative control. If the mean absorbance value of the negative control was lower than 0.105, cutoff value was treated as 0.105. Results were expressed in S/CO value, which was calculated as the ratio of the OD value obtained with the test sample to the cutoff value determined concurrently. The S/CO of 5 or higher was scored as strongly positive reaction; values of 2.1-4.9 as weakly positive and the S/CO of less than 2.1 was scored as negative of humoral immune reaction. Goat anti rabbit IgG-HRP antibody, goat anti human IgG-HRP antibody and goat anti human IgM-HRP antibody were purchased from Bethyl Laboratories, USA.

## Results

### Epitopes stimulating an IgM response in pooled acute sera

**Figure 1:**
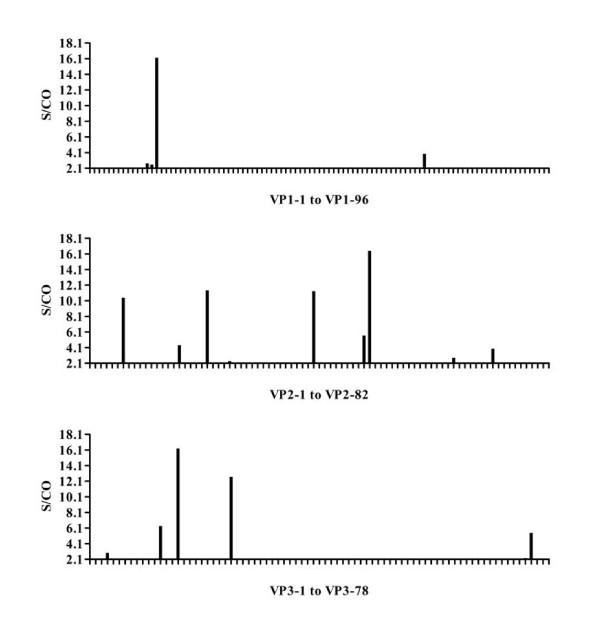
**ELISA results for peptide screening of EV71 structural proteins with pooled acute disease sera**. Eight acute EV71 sera were collected from infected children, and confirmed as EV71 positive by RT-PCR carried out on extracted swab samples. Equal volumes of serum from each infected child were mixed and then 80-fold diluted with saline before used for screening the panel of structural protein synthetic peptides. An indirect ELISA assay was used. The × axis in the histogram shows the synthetic peptides for each structural protein and the y axis indicates the S/CO values.

The ELISA results screening pooled acute disease sera were shown in Figure 1. The majority of human IgM epitopes were mapped to VP2 and VP3 proteins with peptides vp2-6, vp2-21, vp2-40, vp2-50, vp3-10, vp3-15 and vp3-24 all judged as strongly positive reaction. While in VP1 protein, only one peptide (vp1-14) showed a strong response, and another peptide (vp1-70) exhibited a weakly positive reaction (Figure 1).

### Epitope mapping using individual acute stage serum samples

Individual serum of acute stage disease was screened (Table 1). The panel of synthetic peptides that had the S/CO greater than 5 in Figure 1 were recognized by individual acute stage serum. A total of 10 human anti-EV71 IgM epitopes (vp1-14 of VP1;vp2-6, vp2-21, vp2-40 and, vp2-50 of VP2;vp3-10, vp3-12, vp3-15, vp3-24, vp3-75 of VP3) were revealed.

**Table 1:** Tabulated ELISA assay results from screening the panel of EV71 specific synthetic peptides

No. *	Basic information				EV71 Neutralizing antibody titers	EV71 IgM test	VP1-14	VP2-6	VP2-21	VP2-40	VP2-50	VP3-10	VP3-12	VP3-15	VP3-24	VP3-75
			
	Sex	Age (years)	illness onset (days)	PCR dection		OD value	OD value	OD value	OD value	OD value	OD value	OD value	OD value	OD value	OD value	OD value
S1	male	5	3	EV71	384	0.819	1.514	0.643	1.343	0.937	1.117	1.496	0.506	1.190	0.803	0.352
S2	female	4	0	EV71	512	0.897	1.304	0.862	0.971	0.809	1.026	1.322	0.583	0.726	0.809	0.256
S3	male	3	0	EV71	24	0.801	1.293	0.894	0.973	0.665	0.937	0.941	0.483	0.812	0.698	0.249
S4	male	4	2	EV71	640	1.009	0.819	0.427	0.535	0.451	0.604	0.758	0.160	0.426	0.327	0.133
S5	male	2	3	EV71	24	0.150	0.332	0.195	0.206	0.196	0.223	0.285	0.101	0.170	0.133	0.068
S6	male	3	1	EV71	128	0.307	0.834	0.440	0.535	0.430	0.540	0.511	0.280	0.445	0.386	0.187
S7	male	5	1	EV71	512	0.622	0.510	0.258	0.164	0.227	0.363	0.331	0.107	0.224	0.253	0.072
S8	female	1.5	0	EV71	16	0.478	0.721	0.521	0.585	0.457	0.562	0.528	0.321	0.373	0.408	0.212
N1	male	29		/	/	0.032	0.058	0.057	0.048	0.047	0.048	0.054	0.041	0.054	0.038	0.046
N2	male	34		/	/	0.006	0.110	0.085	0.076	0.059	0.086	0.091	0.037	0.072	0.056	0.036
N3	male	30		/	/	0.006	0.180	0.151	0.155	0.125	0.141	0.154	0.107	0.099	0.110	0.089
N4	female	50		/	/	0.016	0.095	0.053	0.044	0.041	0.061	0.065	0.017	0.053	0.033	0.076

* S1-8 are the acute stage sera samples from infected children. N1-N4 is EV71 negative control sera./: negative by PCR detection or by microneutralization assay

### Mapping human anti EV71 IgG epitopes in convalescent sera

**Figure 2:**
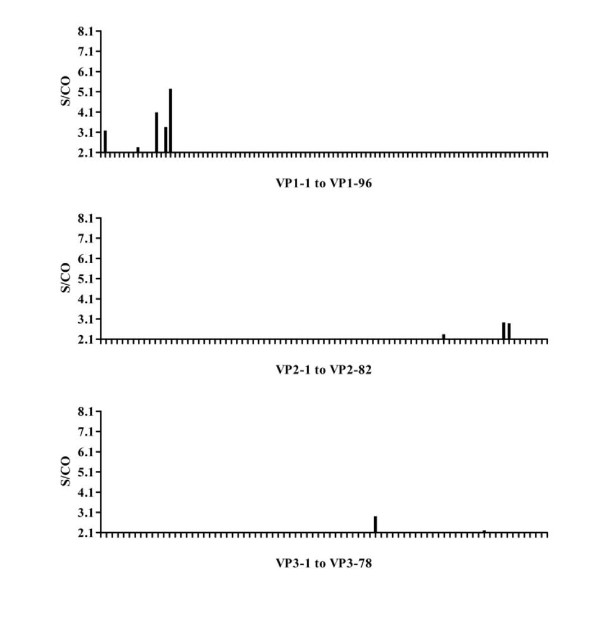
**ELISA results for peptide screening of EV71 structural proteins with pooled convalescent sera**. Convalescent sera were collected from eight children confirmed as EV71 infection by RT-PCR. Equal volumes of serum from each infected child were mixed and then 80-fold diluted with saline before used for screening the panel of structural protein synthetic peptides. An indirect ELISA assay was used. The × axis in the histogram shows the synthetic peptides for each structural protein and the y axis indicates the S/CO values.

The convalescent serum samples from eight infected individuals were pooled and detected by an indirect ELISA assay to map IgG epitopes. The results showed a single strongly positive (vp1-15) and two weakly positive (vp1-12 and vp1-14) IgG epitopes in VP1 peptide. By contrast, only scattered weakly positive results were detected when screening the synthetic peptides of VP2 and VP3 (Figure 2).

### Identification of rabbit anti EV71 IgG epitopes

**Figure 3:**
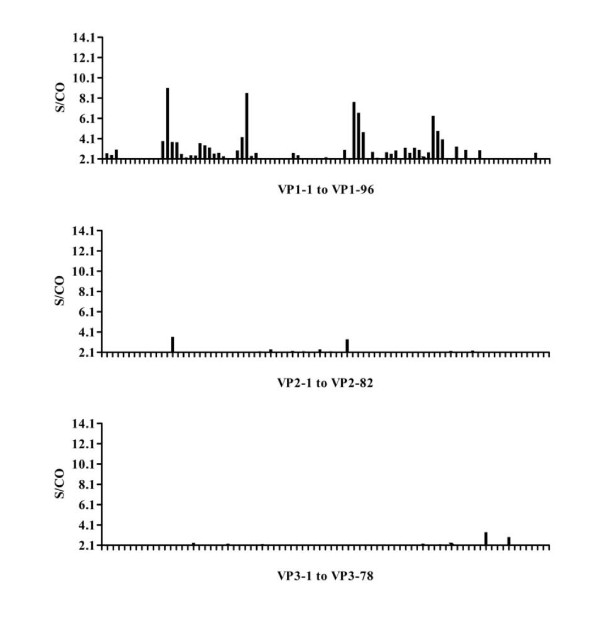
**ELISA results for peptide screening of EV71 structural proteins with rabbit convalescent sera**. Convalescent serum was collected from a rabbit immunized with EV71 virus particles. The serum was 80-fold diluted with saline before used for screening the panel of structural protein synthetic peptides. An indirect ELISA assay was used. The × axis in the histogram shows the synthetic peptides for each structural protein and the y axis indicates the S/CO values.

The panels of synthetic peptides were also screened using a rabbit immunized convalescent sera inactivated by EV71 BJ08 strain. Pre-immunization serum was used as a negative control. The results showed that peptides vp1-14, vp1-31, vp1-54 and vp1-71 in VP1 protein reacted strongly with the sera, while only weakly positive reactions were detected for peptides in VP2 and VP3 (Figure 3).

## Discussion

The structural protein VP1 has usually been considered as an indicator of eliciting a protective immune response after EV71 infection. Consequently, most previous studies aimed at identifying linear humoral epitopes for EV71 were focused on this viral protein. Using 95 overlapping synthetic peptides spanning VP1, Foo, *et al. *found that two peptides, SP55 (amino acids 162-177: PESRESLAWQTATNPC) and SP70 (amino acids 208-222: YPTFGEHKQEKDLEYC) were able to induce neutralizing antibodies against EV71 using an *in vitro *microneutralization assay and a neonate mouse challenge assay [11]_. _A monoclonal antibody (clone 22A12) elicited by SP55 and SP70 immunization was found possessing strong neutralizing activity against EV71 using *in vitro *neutralization assay [18]. Miao, *et al. *developed an EV71/Cox A16-bispecific MAb using VP1 of EV71, which successfully improved a high-quality EV identification kit, and could be used as a diagnostic tool for identifying a wide range of EVs isolated from infected patients [19]. VP2 and VP3 were under certain immune pressure since they are also exposed outside of the EV71 viral capsid. It is necessary to explore epitopes on VP2 and VP3. Liu, *et al. *screened a panel of 153 overlapping synthetic peptides covering the entire sequences of VP1, VP2 and VP3 of EV71. VP2-28 and VP1-43 were recognized by neutralizing antisera (IgG) generated from rabbit and murine, respectively [20].

Over the past decade, EV71 outbreak has frequently occurred in the Asia-Pacific [2-8]. It is the urgent to understand human IgM and IgG epitopes in order to provide the basis for the development of vaccines and diagnostic reagents. In the current study, the strongest reaction to VP1 peptide vp1-15 was obtained in convalescent sera from infected children. The immunized rabbit sera showing positive epitopes reaction were also primarily located on VP1 protein. The peptide vp1-14 had the strongest reaction, followed by gradually decreased reactive peptides vp1-31, vp1-54 and vp1-71. The positive reaction in various degrees for immunized rabbit sera and convalescent human sera may reflect different levels of virus infection and inactivated virus immunization. Overall, these results were consistent with the previous reports [11,13,20-22].

Detecting an IgM response is important for early diagnosis of infectious diseases [16,19,23]. However, no IgM epitopes have been identified for EV71 infection to date. Although VP1 contains the major identified epitopes, VP2 and VP3 located on the outside of the viral particle may also be associated with a protective immune response occurred upon or after infection. Consistent with this assumption, the current study identified several clearly reactive epitopes in VP2 and VP3 although their reactions were not so strong. Ten human EV71 IgM epitopes were identified for the first time, and all identified peptides were positively reacted to 8 tested sera, which indicated that EV71 infection may consistently induce multi-epitope anti EV71 IgM response.

Comparison the distribution of immune-reactive IgM and IgG epitopes, our data showed that IgM epitopes were mainly located in VP2 and VP3 capsid proteins, while only one peptide (vp1-14) was located in VP1 eliciting a strong IgM reaction. Another peptide (vp1-15) located in VP1 protein evoked a strong IgG response (Table 2).

**Table 2:** Immunodominant linear epitopes identified by EV71 antisera

anti-EV71 epitopes	Residues	Peptide sequences
human anti-EV71 IgM epitopes		
vp1-14	40-51aa	DTGKVPALQAAE
vp2-6	16-27aa	LTIGNSTITTQE
vp2-21	61-72aa	NRFYTLDTKLWE
vp2-40	118-129aa	HQGALLVAVLPE
vp2-50	148-159aa	YKQTQPGADGFE
vp3-10	28-39aa	FHPTPCIHIPGE
vp3-12	34-45aa	IHIPGEVRNLLE
vp3-15	43-54aa	LLELCQVETILE
vp3-24	70-81aa	RFPVSAQAGKGE
vp3-75	223-234aa	NFTMKLCKDASD
human anti-EV71 IgG epitopes		
vp1-15	43-54aa	KVPALQAAEIGA
rabbit anti-EV71 IgG epitopes		
vp1-14	40-51aa	DTGKVPALQAAE
vp1-31	91-102aa	GEIDLPLEGTTN
vp1-54	160-171aa	APKPDSRESPAW
vp1-71	211-222aa	FGEHKQEKDLEY

In conclusion, our data, for the first time, mapped human anti EV71 IgM and IgG epitopes to the VP1, VP2 and VP3 capsid proteins. These results are valuable for the evaluation of EV71 vaccine immunogenicity and the development of early diagnostic reagents.

## Abbreviations

EV71: Enterovirus 71; ELISA: Enzyme-linked immunosorbent assay; IgM: Immunoglobulin M; IgG: Immunoglobulin G; HFMD: Hand foot and mouth disease; RNA: Ribonucleic acid; SPF: Specific pathogen Free; PCR: Polymerase chain reaction; CA16: Coxsackievirus A16; CDC: Centers for disease control; PBST: Phosphate buffered saline containing 0.05% Tween-20; HRP: Horseradish peroxidase; TMB: 3,3',5,5'-Tetramethylbenzidine; OD: Optical densities; S/CO: Sample optical densities value/Cutoff value; USA: United States of America.

## Competing interests

The authors declare that they have no competing interests.

## Authors' contributions

GF and WYP carried out the laboratory experiments, analyzed the data, interpreted the results and drafted the manuscript. YX and MQY performed molecular genetic studies and sequence alignment. ZFC contributed to specimen collection and clinical diagnosis. LZL and GF participated in the design of the study and performed the statistical analysis. WJZ, LFX, LZL, ZFC, LFM, YJY, LS conceived of the study, and participated in its design and coordination and helped to draft the manuscript. All authors read and approved the final manuscript.
